# A categorical network approach for discovering differentially expressed regulations in cancer

**DOI:** 10.1186/1755-8794-6-S3-S1

**Published:** 2013-11-11

**Authors:** Nikolay Balov

**Affiliations:** 1 Department of Biostatistics and Computational Biology, University of Rochester Medical Center, Rochester, NY-14642, USA

## Abstract

**Background:**

The problem of efficient utilization of genome-wide expression profiles for identification and prediction of complex disease conditions is both important and challenging. Polygenic pathologies such as most types of cancer involve disregulation of many interacting genes which has prompted search for suitable statistical models for their representation. By accounting for changes in gene regulations between comparable conditions, graphical statistical models are expected to improve prediction precision.

**Methods:**

In comparison problems with two or more experimental conditions, we represent the classes by categorical Bayesian networks that share one and the same graph structure but have class-specific probability parameters. The graph structure is learned by a score-based procedure that maximizes the difference between class probabilities using a suitable measure of divergence. The proposed framework includes an indirect model selection by adhering to a principle of optimal class separation and identifies interactions presenting significant difference between the compared conditions.

**Results:**

We evaluate the performance of the new model against some benchmark algorithms such as support vector machine, penalized linear regression and linear Gaussian networks. The classifiers are compared by prediction accuracy across 15 different data sets from breast, lung, gastric and renal cancer studies. In addition to the demonstrated strong performance against the competitors, the proposed method is able to identify disease specific changes in gene regulations which are inaccessible by other approaches. The latter is illustrated by analyzing some gene interactions differentiating adenocarcinoma and squamous cell lung cancers.

## Introduction

High-throughput technologies such as microarrays supply means for genome-wide observation on cell samples and provide unique opportunities for studying complex heterogeneous diseases. It is understood for example that the highly polygenic pathology of cancers involves not single gene mutations but alternations in multiple genetic pathways [1]. Even cancer subtypes with a common origin can be driven by very different disregulations on gene interaction level [2]. Computational analysis of high-throughput genetic data thus requires adequate multivariate statistical models with capacity of studying gene regulations at system level. Graphical models such as Bayesian networks have been proposed for describing cell signaling processes [3] and analysis of expression data [4], to mention but a few, and have been accepted as important tools in the field of systems biology.

We present a categorical Bayesian network framework based on an original learning method for analysis of gene expression data, in particular, for classification of gene expression profiles coming from different populations. Typical applications include diagnostic tests for disease conditions and differentiating between disease subtypes. More formally, we assume we are given a sample of *n *microarrays measuring the expression level of *N*, potentially thousands, genes or gene probes under two different experimental conditions. Usually *n *is much smaller than *N*. We are interested in designing a methodology for setting apart these two conditions and be able to designate gene profiles to their originating classes.

Many classical approaches such as linear discriminant analysis are ill suited for large *N *small *n *settings. Other models, such as LASSO [5] and support vector machines (SVM) [6], either disregard possible gene associations or defy explicit interpretation. In contrast, Bayesian network (BN) models are able to identify associated genes and parsimoniously describe the global gene interaction structure [4,7,8]. BNs have been recognized as worthwhile alternative to more traditional state-of-art models in terms of discrimination and classification power [9,10], but their widespread application is nevertheless not evident.

A major issue in applying BNs to analysis of gene expression data is choosing the complexity of the underlying graph structure. Simple models may undermine the complexity of the observed gene system. On the other hand, too complex ones often overfit the data and, as a result, diminish the prediction power. A standard approach addressing this model selection problem employs the Bayesian paradigm and performs maximum a posteriori (MAP) estimation [11]. Since the posterior is usually not available in closed form, the MAP approach needs to be implemented by computationally expensive Monte Carlo procedures [9] or by applying some heuristic algorithms that approximate MAP [10]. Moreover, the efficiency of MAP in the context of model selection crucially depends on the selected prior. The so called constrained-based learning methods such as the PC algorithm [12] also require setting additional parameters (the α-level for the conditional independence tests in PC) in order to choose the 'right' complexity of the model. Similarly, the score-based learning methods such as the penalized maximum likelihood estimation [13] rely on parameters controlling the penalization as a function of complexity. Therefore, in single-population(class) settings, model selection seems to involve inevitably some external, outside the data itself, input or control.

In the theory of statistical learning there are two standard approaches for choosing model selection parameters. One is based on large sample asymptotic properties of the estimator and ensures that the latter is consistent. The other accepted practice is to follow a data-driven cross-validation (CV) procedure. Both approaches however have disadvantages: the former one may suffer from lack of optimality in finite sample size settings, while the CV approach can be computationally prohibitive for the purpose of network learning. In addition, some authors have raised questions on the theoretical justification of CV [14]. Our approach is motivated by the intuitive expectation that in two(multi)-class problems, model selection can be more easily resolved in a self-contained manner. We propose a categorical BN framework with a score-based learning algorithm that includes the class membership information in the optimization function. It addresses the model selection problem by choosing networks that provide optimal class separation. Our methodology can be applied to gene expression data of reasonable size and, as we show, is not only effective in gene profile classification, but can provide important insights on the functionality of the observed biological systems.

**Figure 1:**
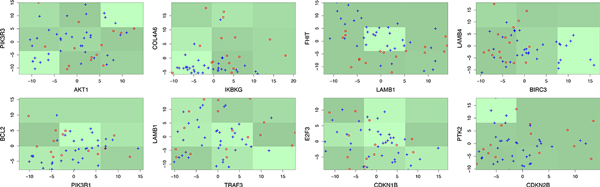
**Example of gene expression discretization and 3-nomial representation of gene interactions**. Shown are 8 pairs of genes from the *Small Cell Lung Cancer *pathway and observations from LNG1 data set. The class membership of the points is indicated in red and blue. Overlaid on the cross plots are the discretization regions shaded according to the KL-values $P_{ij}\text{log}\left(P_{ij}/P_{ij}^{0}\right),\;\;i,j=1,2,3$ (regions with higher values are shown lighter).

Categorical Bayesian networks (CBNs) represent associations between discrete random variables through directed acyclic graphs. In contrast to linear Gaussian BNs, CBNs are capable of representing non-linear relationships between their node-variables. Although the application of CBNs to continuous gene expression data involves loss of information in the necessary process of discretization, often (see Figure 1 below for examples), CBNs benefit from more faithful representation of the observed gene interactions than linear BNs. A number of existing methods exploit CBNs to mitigate gene expression noise and improve classification accuracy [15,16]. In the context of microarray data, another advantage of discretization is its robustness to the so-called lab or batch effect inherent in many multi-laboratory studies [17].

The paper is organized as follows. We start with a brief introduction to CBNs, the Maximum Likelihood (ML) principle for CBN estimation and formulate a novel scoring function as alternative to the standard log-likelihood function used in ML. Our discriminating function is based on the Kullback-Leibler (KL) divergence between conditional probability tables (Eq. (3) below). For given two-class training data, we reconstruct a CBN that includes only those gene connections that present significant class differences and thus reveal implicated gene interaction changes. We then describe a classification algorithm that models the observed conditions using the already estimated graph structure. The representing CBNs are distinguished by their class-specific probability tables. As usual, the class assignment of new observations is based on the likelihoods of the estimated class CBNs.

In the Results section, the proposed method is evaluated on 15 microarray data sets - 6 breast cancer, 3 lung cancer, 3 gastric cancer and 3 renal cancer studies - grouped in pairs by phenotypic and class criteria. The performance of 4 algorithms - the proposed one, SVM, LASSO and a linear Gaussian BN classifier based on the PC algorithm for structure learning - are compared using sets of differentially expressed genes as well as on a collection of gene pathways from the KEGG database. Compatible but different data sets are chosen as (*training, test*) pairs for evaluation. The proposed classifier demonstrates favorable prediction performance across the selected data set pairs. Finally, we illustrate the analytical and interpretation merits of our methodology by focusing on the lung cancer data sets and inspecting some regulations that have significant role in distinguishing adenocarcinoma from squamous cell lung cancers.

## Methods

Our methodology was first introduced in [18]; below we presented it in some more details.

In regard to the notation, we shall use capital letters to denote model parameters and random variables, and small ones for the realizations of these variables. Let *X_i_, i *= 1,.., *N *be random categorical variables. Categorical Bayesian network (*G, P*) with nodes *X_i_*'s is a probability model based on directed acyclic graph (DAG) *G *and conditional probability table *P *defined as follows. *G *can be described by a collection of sets *Pa_i_*'s such that for each *i, Pa_i_*, called parent set of *X_i_*, comprises all *X_j_*'s for which there is a directed edge connecting *X_j _*and *X_i _*in *G*. We shall use index *k *to denote the categorical levels of *X_i _*and multi-index *j *for the combination of parent states of *X_i_*, and with slight abuse of notation shall write *k *∈ *X_i _*and *j *∈ *Pa_i_*. The second component of a CBN is the table *P *of conditional probabilities *Pr*(*X_i_*|*Pa_i_*). Let *P_i,kj _*≡ *Pr*(*X_i _= k*|*Pa_i _= j*). For each *i *and *j*, the multinomial distribution of (*X_i_*|*Pa_i _= j*) is defined by the probability vector $P_{i,j}\equiv {\left(P_{i,kj}\right)}_{k\in X_{i}},\sum_{k\in X_{i}}P_{i,kj}=1$. Then we have $P={\left\{P_{i,j}\right\}}_{i,j}$.

With [*X_i_*] and [*Pa_i_*] we shall denote the number of states of *X_i _*and *Pa_i_*, respectively, and with |*Pa_i_*| the number of parents of *X_i_*. Clearly, $\left[Pa_{i}\right]=\prod_{X_{a}\in \;\;Pa_{i}}\left[X_{a}\right]$. The complexity of the CBN (*G, P*) is given by $df\left(G\right)=\sum_{i-1}^{N}\left[Pa_{i}\right]\left(\left[X_{i}\right]-1\right)$ and equals the degree of Freedom for defining the probability table *P*.

For any DAG *G*, there is a node order Ω, also called causal order, such that the parents of each node always precede that node in Ω, a fact that we write symbolically as *X*_Ω(1) _≺ *X*_Ω(2) _≺... ≺ *X*_Ω(*N*)_. Formally, Ω is a permutation of the indices 1,..., N, such that for any *i *> 0, *Pa*_Ω(*i*) _⊂ {*X*_Ω(1)_, *X*_Ω(2)_, ..., *X*_Ω(*i*−1)_}. For any order Ω, with G(Ω) we shall denote the class of DAGs that are compatible with Ω.

### Learning categorical Bayesian networks from two-class data

We approach CBN learning from the perspective of classification problems where the observations are assumed to come from two different classes. We describe an algorithm that utilizes the class label information to find a DAG attaining maximum class discrimination with respect to a suitable measure. The essential component of our method is the graph structure estimation since the optimal conditional probability table can be easily inferred for any given DAG.

Let ${\left\{x^{s}\right\}}_{s=1}^{n}$ be a *n*-sample of independent observations on ${\left\{X_{i}\right\}}_{i=1}^{N}$ and let each observation *x^s ^*have a label *c^s ^*∈ {0, 1} that assigns it to one of the two classes; *c^s^*'s are assumed to be observations on a binary random variable *C*. We denote the labeled sample with $D_{n}={\left\{\left(x^{s},c^{s}\right)\right\}}_{s=1}^{n}$.

The log-likelihood function of a CBN (*G, P*) with nodes ${\left\{X_{i}\right\}}_{i=1}^{N}$ with respect to the unlabeled sample ${\left\{x^{s}\right\}}_{s=1}^{n}$ is

(1) $$l\left(G,P|D_{n}\right)=\overset{N}{\underset{i=1}{\sum }}\underset{j\;\in \;Pa_{i}}{\sum }\underset{k\;\in \;X_{i}}{\sum }n_{i},k_{j}\text{log}P_{i,k_{j}},$$

where $n_{i},k_{j}\equiv \sum_{s=1}^{n}1_{\left\{x_{i}^{s}=k,pa_{i}^{s}=j\right\}}$ and $n_{i,j}\equiv \sum_{k}n_{i},k_{j}$. Note that for each *i *we have $\sum_{j\in \;Pa_{i}}n_{i,j}=n$. For a fixed DAG *G*, the MLE $\hat{P}$ of *P *is obtained by maximizing *l*(*G, P*|*D_n_*) as a function of *P*

(2) $$l\left(G|D_{n}\right)\equiv \underset{P}{\text{max}}l\left(G,P|D_{n}\right)=\overset{N}{\underset{i=1}{\sum }}\overset{N}{\underset{j\in Pa_{i}}{\sum }}n_{i,j}\overset{N}{\underset{k\in X_{i}}{\sum }}{\hat{P}}_{i,k_{j}}\text{log}{\hat{P}}_{i,k_{j}},$$

where ${\hat{P}}_{i,k_{j}}\equiv n_{i,kj}/n_{i,j}$ is the point estimate of *P_i,kj_*. It is a well known fact that by increasing the complexity of *G*, that is, by adding new edges in *G*, the likelihood (2) can only increase. Therefore the MLE solution for *G *based on (1) will tend to overfit the training data. The latter can be overcome if, instead of the log-likelihood, one optimizes a scoring function of the form *l*(*G*|*D_n_*) − λ*_n_df *(*G*) [13], where λ*_n _*is a penalization parameter indexed by the sample size *n*. Some standard choices include BIC, λ*_n _*= 0.5*log*(*n*)/*n*, and AIC, λ*_n _*= 1/*n*, however, no 'universally' optimal penalization criterion is available. As we have already commented in the introduction, data-driven approaches for selecting λ*_n _*such as CV are not necessarily optimal and their efficiency in two-class discrimination problems is unclear. In contrast, our approach is based on a scoring function, very similar to the likelihood ratio test, that can be used to learn an optimal graph structure without involving additional penalization parameters.

Similarly to *P_i_,k_j_*, let us define the conditional probabilities pertaining to the first experimental class, $P_{i,kj}^{0}=Pr\left(X_{i}=k|Pa_{i}=j,C=0\right)$ and let ${\hat{P}}_{i,kj}^{0}$ be the corresponding point estimators as in (2), that is, ${\hat{P}}_{i,kj}^{0}=n_{i,kj}^{0}\text{/}n_{i,j}^{0}$, where $n_{i,kj}^{0}\equiv \sum_{s=1}^{n}1_{\left\{x_{i}^{s}=k,pa_{i}^{s}=j,c^{s}=0\right\}}$. We then consider the statistics

(3) $$R\left(G|D_{n}\right)=\frac{1}{n}\overset{N}{\underset{i=1}{\sum }}\underset{j\in \;Pa_{i}}{\sum }\underset{k\in \;X_{i}}{\sum }n_{i,kj}\text{log}\left({\hat{P}}_{i,kj}/{\hat{P}}_{i,kj}^{0}\right)$$

and introduce the scoring function

(4) $$S\left(G|D_{n}\right)=\frac{R\left(G|D_{n}\right)}{df\left(G\right)},$$

the intuition behind which is given below. Given a collection  G of DAGs with nodes ${\left\{X_{i}\right\}}_{i=1}^{N}$, we propose the following estimator of *G*

(5) $$\overset{\text{\textasciicircum{}}}{G}=arg\underset{G\in G}{\text{max}}S\left(G|D_{n}\right)$$

which we shall tentatively refer to as BNKL estimator.

Equivalently,  R can be expressed as

$$R\left(G|D_{n}\right)=\frac{1}{n}\overset{N}{\underset{i=1}{\sum }}\underset{j\;\in \;Pa_{i}}{\sum }n_{i,j}d_{K\;L}\left({\hat{P}}_{i,j}||{\hat{P}}_{i,j}^{0}\right),$$

where *d_K L _*denotes the Kullback-Leibler (KL) divergence between the multinomial distributions ${\hat{P}}_{i,j}={\left\{{\hat{P}}_{i,kj}\right\}}_{k}$ and ${\hat{P}}_{i,j}^{0}={\left\{{\hat{P}}_{i,kj}^{0}\right\}}_{k}$. The optimization problem (5) aims at finding a DAG that achieves maximum class separation with respect to the accumulated KL-divergence between the node conditional probability tables. Note that we always have $R\left(G|D_{n}\right)\geq 0$. Moreover, if ${\hat{P}}_{i,j}^{0}$ are uniform distributions, that is, ${\hat{P}}_{i,j}^{0}={\left(1/\left[X_{i}\right]\right)}_{i=1}^{\left[X_{i}\right]}$, then (3) reduces to (1) up to an additional constant due to the equality

$$\frac{1}{n}\overset{N}{\underset{i=1}{\sum }}\underset{j\in \;Pa_{i}}{\sum }n_{i,j}d_{K\;L}\left({\hat{P}}_{i,j}||{\hat{P}}_{i,j}^{0}\right)=\frac{1}{n}l\left(G|D_{n}\right)+\overset{N}{\underset{i=1}{\sum }}\text{log}\left(\left[X_{i}\right]\right).$$

We can therefore look at $R\left(G|D_{n}\right)$ as an extension of the maximum log-likelihood *l*(*G*|*D_n_*) to two-class problems.

For a fixed DAG *G*, the statistics 2nR is in fact equivalent to the likelihood ratio chi-squared statistics (also known as *G*^2 ^statistics) applied to $n\hat{P}$ and $n{\hat{P}}^{0}$ viewed as observed and expected counts, respectively. Not surprisingly then, under the null hypothesis $H_{0}:P_{i,j}=P_{i,j}^{0}$, for all *i, j*, $2n\kappa R\left(G|D_{n}\right)$ is asymptotically *χ*^2 ^distributed with *df *(*G*) degree of freedom, where *κ = Pr*(*C *= 0)/*Pr*(*C *= 1) is the odds ratio for the first class (the formal proof of this fact is out of the scope of this article).

The role of the factor *df*(*G*) in the denominator of (5) is to assist model selection. From information-theoretical perspective, *df*(*G*) represents the amount of memory required for saving all of the states of a CBN with DAG *G*. Since $R\left(G|D_{n}\right)$ measures the class differences with respect to *G*, we can think of the scoring function (4) as an estimate of the degree of class separation per unit complexity. Let R(G) be the population version of (3) obtained by replacing $\hat{P}$ with the population probabilities *P*, that is

$$R\left(G\right)=\overset{N}{\underset{i=1}{\sum }}\underset{j\in \;Pa_{i}}{\sum }Pr\left(Pa_{i}=j\right)d_{K\;L}\left(P_{i,j}||P_{i,j}^{0}\right).$$

We say that *G*_0 _achieves most efficient class separation in  if

(6) $$G_{0}=arg\underset{G\in G}{\text{max}}\frac{R\left(G\right)}{df\left(G\right)}.$$

Then, provided that *G*_0 _is unique maximizer, it can be easily shown that $\hat{G}$ is a consistent estimator of *G*_0_, a claim that makes (5) a sound statistical procedure.

We proceed into some computational aspects of problem (5). Because $S\left(G|D_{n}\right)$ is usually highly non-regular function (non-smooth and non-convex), finding the optimal DAG essentially requires an exhaustive search in  G. In order to make the problem computationally manageable we thus need to apply some strong restrictive conditions on  G. First, we assume that the parent sizes are bounded above by a constant *M*. The value of *M *should depend on the samples size *n *used for estimation and we do not recommend *M *> 2 unless n is in the hundreds. Second, we limit the search in (5) to DAGs compatible with a fixed but optimally chosen node order. An intuitive causality argument suggests that if we believe that node conditional distributions are set rather independently of each other than otherwise, for a regulation *X*_1 _→ *X*_2_, it seems more plausible for *X*_2 _to have higher between-classes marginal difference than *X*_1_. If we accept this argument, we would be inclined to assume that:

(7) nodeswithlowermarginalclassdifferenceareupstreamtheDAGofthenetwork;

where 'upstream' is understand as earlier in the node order of the DAG. In the context of gene regulations we periphrase this principle in 'target' and 'biomarker' terminology as follows: 'target' genes present lower differential expression in comparison to the 'biomarker' genes and are thus situated upstream the regulation network with respect to the latter. Hereafter we refer to an order satisfying (7) as order of increasing differential expression or IDE.; see Algorithm 1 below for its estimation.

Formally, for the purpose of solving (5), we consider collections of DAGs of the form

(8) $$G\left(\text{Ω},M\right)=\left\{G|X_{\text{Ω}\left(1\right)}≺X_{\text{Ω}\left(2\right)}≺\ldots ≺X_{\text{Ω}\left(N\right)},|Pa_{i}|\;\leq \;M,\forall i\right\},$$

where Ω satisfies (7). In the actual data analyses below we use *M *= 2 as a compromise between degree of network connectivity and computational complexity. For classes G(Ω,M), the optimal DAG $\;\hat{G}$ can be found by an efficient exhaustive search with polynomial complexity. In fact, BN estimation restricted to type (8) classes of DAGs is not new and can be traced back to [19]. The BNKL algorithm is implemented in the *sdnet *package for **R**, [20]. Below, we present average times of BNKL estimation of random CBNs with different sizes *N*, 3 categories per node, maximum parent size M = 2 and sample size *n *= 250 (Table 1).

**Table 1:** 

*N*	25	50	100	250	500
time(sec)	0.01	0.06	0.53	16	417

The computational times are concordant with the theoretical complexity of the algorithm *O*(*nN*^*M *+ 1^).

### A network model for classification of gene expression profiles

We return to the main goal of this investigation - developing a CBN-based classifier for two-class problems. We have shown, Eq. (5), how we can choose a graph structure that achieves optimal separation of a labeled sample. We use the estimated structure as a common DAG of two CBNs that model the two classes with distinct probability tables. This approach, 'one DAG, two probability tables', has been previously adopted by other BN-based classifiers [21].

Gene expression data is acquired by a multi-stage process the result of which are continuous variables representing the expression levels of pre-specified gene probes. Since CBN is a discrete model, the initial step in our inference framework involves discretization - any sample ${\left\{y^{s}\right\}}_{s=1}^{n}$ of observations on the gene-nodes ${\left\{X_{i}\right\}}_{i=1}^{N}$ is transformed into categorical sample ${\left\{x^{s}\right\}}_{s=1}^{n}$. Gene expression levels are often discretized into 3 categories - 'under-expressed', 'baseline' and 'over-expressed' [16]. Although more sophisticated procedures are certainly possible, in our experiments we employ a 3-level uniform discretization as follows. After excluding 5% of the most extreme values, a standard precaution against outliers, the range of *y*'s is divided into equal intervals and an observation *y *is assigned a categorical value *x *according to the interval into which *y *falls. The uniform discretization is simple to implement and have good performance in practice. We emphasize that, as should be the case in all well designed *training *→ *test *prediction studies, the discretization parameters (cut-off points) are determined strictly from the training sample and are used to discretize the test sample.

More formally, we assume that: (i) the class samples *D*_0 _= *D *∩ {*c *= 0} and *D*_1 _= *D *∩ {*c *= 1} come from two CBNs, (*G*_0_, *P*^0^) and (*G*_0_, *P*^1^), with DAG *G*_0 _and probability tables *P*^0 ^and *P*^1^; (ii) *G*_0 _is efficient in sense of (6); (iii) *G*_0 _is compatible with an IDE order Ω and has a maximum parent size of *M*. Since *G*_0 _is unknown in advance, the assumptions (ii) and (iii) cannot be checked. Instead, (ii) and (iii) should be considered technical assumptions specifying the properties of the estimated networks. All prerequisites being set, we propose Algorithm 1: the first part of it estimates *G*_0_, *P*^0 ^and *P*^1^, while the second one performs classification of test samples.

**Algorithm 1 **BNKL Classification

1. **Training**. Input: continuous labeled training sample ${\left\{\left(y^{s},c^{s}\right)\right\}}_{s=1}^{n}$.

(a) *Node order estimation, IDE *(7): For each *i*, perform t-test on *y_i_*'s comparing the 2 classes. Set $\hat{\text{Ω}}$to be the order of decreasing (t-test) p-values.

(b) *Uniform discretization*: For each *i*, set *µ_ik _= q*_1 _+ *k*(*q*_2 _− *q*_1_)/3, *k *= 1, 2, where *q*_1 _and *q*_2 _are the 2.5 and 97.5 percentiles of the training observations *y_i_*'s. Discretize *y_i_*'s into 3 categories using the cut-off points *µ*_*i*1 _and *µ*_*i*2_.

(c) Find the optimal DAG $\;\hat{G}$ in $G\left(\hat{\text{Ω}}\right)$ according to Eq. (5).

(d) Define CBNs $\left(\hat{G},{\hat{P}}^{0}\right)$ and $\left(\hat{G},{\hat{P}}^{1}\right)$ by estimating the class-specific conditional probability tables ${\hat{P}}^{0}\left(\hat{G}|D_{0}\right)$ and ${\hat{P}}^{1}\left(\hat{G}|D_{1}\right)$ as in Eq. (2).

2. **Prediction**. Input: continuous test observation *z*.

(a) For each *i*, discretize $z_{i}\mapsto x_{i}$ using the training cut-off points *µ*_*i*1 _and *µ*_*i*2_.

(b) Calculate the log-likelihoods $l_{0}=l\left(\hat{G},{\hat{P}}_{0}|x\right)$ and $l_{1}=l\left(\hat{G},{\hat{P}}_{1}|x\right)$ according to Eq. (1).

(c) Assign *z *to the class with greater log-likelihood *l*.

To avoid numerical instabilities in Algorithm 1, all zero slots in the estimated conditional probabilities ${\hat{P}}^{0}$ and ${\hat{P}}^{1}$ are reset to a minimum positive value of 1/(3*n*) (the resolution of a training sample of size *n *to populate a 3-nomial distribution) and then re-normalized so that $\sum_{k}{\hat{P}}_{i,kj}=1$, for all *i *and *j *∈ *Pa_i_*.

Figure 1 shows some examples of gene expression discretization and representation of gene interactions with 3-nomial distributions. For instance, the probabilities *P_ij _= Pr*(*X*_*AKT*1 _= *i*|*X*_*PIK*3*R*3 _= *j*) of the regulation AKT1→PIK3R3 are *P*_*i*1 _= (0.4, 0.4, 0.2), *P*_*i*2 _= (0.23, 0.54, 0.23) and *P*_*i*3 _= (0.17, 0.42, 0.41). The probabilities corresponding to the first class only are $P_{i1}^{0}=\left(0.14,0.72,0.14\right)$, $P_{i2}^{0}=\left(0.28,0.14,0.57\right)$ and $P_{i3}^{0}=\left(0.25,0.5,0.25\right)$. A formal test for the linear association between the two genes fails to detect significant class difference (p-val = 0.18). The relatively large KL-divergence score between *P *and *P*^0 ^however, indicates significant class difference (p-val≈0). The examples also illustrate different types of regulations such as activation (PIK3R3→AKT1, LAMB1→TRAF3) and inhibition (FHIT→LAMB1). The BNKL model, recall, is designed to detect changes in the interactions. For example, FHIT→LAMB1 is apparently inhibitory for the second class (in blue) but neutral for the first (in red) and BNKL perceives that difference.

### Benchmark classifiers

To evaluate the performance of the BNKL algorithm we compare it to 3 established in practice classification methods. We consider SVM with Gaussian kernel as implemented in the *e1071 *package for **R**. The kernel parameter *γ *is tuned via CV on the training data for optimality. The benchmark performance of SVM is well established [22]. Our second choice is LASSO, an algorithm based on *l*_1_-penalized linear regression that is applied as follows. The expectation of the binary class variable is assumed to be a linear combination of a given set of gene-covariates. Then LASSO selects a subset of significant predictor genes using a *l*1-norm-based penalization criteria and discard the rest. The sum of squared errors is used as classification criteria. We use an implementation of the algorithm provided by the *lars *package for **R**.

The third reference classifier, PC, employs a linear BN model as follows: (1) a DAG $\;\hat{G}$ is fitted to the combined sample *D*_0 _∪ *D*_1 _using the PC algorithm [23] with Gaussian test for conditional independence at *α*-level 0.05 (see *pcalg *package for **R**); thus a parent set *Pa_i _*is selected for each i; (2) for each *i*, two distinct sets of (*Y_i_*|*Pa_i_*)-regression parameters are estimated for each class separately; (3) test samples are classified according to the conditional likelihoods. Note that, SVM, LASSO and PC, in contrast to BNKL, are applied directly to continuous observations on ${\left(X_{i}\right)}_{i=1}^{N}$.

## Results

This section complements the preliminary results presented in [18] and provides a comprehensive evaluation of BNKL on a diverse collection of gene expression data. We consider 15 samples stratified in 5 groups by cell type and disease condition. Table 2 provides a detailed description of the microarrays including their reference number, platform and class sample sizes. We have 2 breast cancer data sets with subjects grouped by survival status and 4 other using estrogen receptor (ER) status as classification criteria. Also considered are 3 lung cancer data sets comparing adenocarcinoma and squamous cell carcinoma, as well as, 3 gastric and 3 renal cancer related samples of disease vs. control. All expression data sets are obtained from the Gene Expression Omnibus (GEO, http://www.ncbi.nlm.nih.gov/geo/) and prior to the classification analysis are pre-processed by applying the following three steps. First, the raw expression sets are normalized using the RMA procedure [24]. Then, the probe expression levels across sample records are standardized. If ${\left\{y_{i}^{s}\right\}}_{s=1}^{n}$ are the expression levels of the *i*-th probe, the standardization is performed according to the formula ${\tilde{y}}_{i}^{s}=\sqrt{n}\left(y_{i}^{s}-\mu_{i}\right)/sd_{i}$, where *µ_i _*and *sd_i _*are the sample mean and standard deviation of $y_{i}^{s}$'s. Standardization is intended to account for some gross disparities in the expression levels of probes coming from different data sets which cannot be handled by the normalization procedure. The latter is especially true for microarrays produced on different platforms such as KDN2 and KDN3. Finally, for each pair considered for across data set classification, we perform consolidation by sub-setting to a common set of gene probes. Again, this step is needed in order to be able to compare between different platforms; for example, while GPL570 can accommodate up to 54675 probes, GPL96 is limited to 22283 probes. For brevity, hereafter we shall refer to gene probes simply as genes.

**Table 2:** Gene expression data sets used in the study

Name	GEO Ref	Platform	Disease Condition	Class Criteria	Samples by Class	Reference
BRS1	GSE1456	GPL96	Breast	survival status	119+40	[25]
BRS2	GSE3494	GPL96	Cancer		181+55	[26]

BER1	GSE2990	GPL96	Breast	ER status	34+149	[27]
BER2	GSE7390	GPL96	Cancer	neg. vs. pos.	64+134	[28]
BER3	GSE20711	GPL570			45+42	[29]
BER4	GSE2034	GPL96			77+209	[30]

LNG1	GSE10245	GPL570	Lung	adenocarcen.	18+40	[31]
LNG2	GSE18842	GPL570	Cancer	vs.	32+14	[32]
LNG3	GSE31799	GPL14189		squamous cell	20+29	[33]

GST1	GSE33335	GPL5175	Gastric	tumor	25+25	[34]
GST2	GSE27342	GPL5175	Cancer	vs. normal	80+80	[35]
GST3	GSE37023	GPL96			112+39	[36]

KDN1	GSE15641	GPL96	Clear Cell	disease	32+23	[37]
KDN2	GSE17818	GPL9101	Renal Cancer	vs. control	102+13	[38]
KDN3	GSE22316	GPL10175			70+13	[39]

We carry out two classification strategies by applying the considered algorithms on two categories of gene subsets: (1) differentially expressed (DE) genes and (2) a collection of curated gene pathways. Below we give more details on these two approaches. The algorithms' performance is evaluated by across data set prediction for pairs of compatible data sets. The first prediction score we use is the balanced accuracy given by ACC = 0.5(TP/P + TN/N), where P and N are the number of test observations in the two classes, while TP and TN are the number of correctly assigned observations to the first and second class, respectively. The 'random guess' procedure thus has accuracy of 0.5 on average and so does any algorithm that assigns all observations to one class. As a second criteria we employ the area under the curve between sensitivity TPR = TP/(TP+FN) and FPR = TP/(TP+FN), known as AUC. An AUC of 1 represents perfect class separation.

Tables 3, 4 and 5 present prediction results for 16 compatible data set pairs. We calculate the pairwise ACC and AUC scores as follows. For a pair (*A, B*) with sample sizes *n*_A _and *n*_B _respectively, we perform the classifications *A *→ *B *(*A *training, *B *test) and *B *→ *A *(*B *training, *A *test), and calculate the corresponding ACC and AUC scores. Then we report the overall scores by weighting the individual scores according to the test sample sizes,

**Table 3:** Prediction performance using top 100 DE genes.

		ACC				AUC		
**datasets**	**BNKL**	**SVM**	**LAS**	**PC**	**BNKL**	**SVM**	**LAS**	**PC**

BRS1, BRS2	0.62	0.53	0.55	0.52	0.68	0.60	0.66	0.61

BER1, BER2	0.79	0.71	0.75	0.63	0.89	0.82	0.90	0.87
BER2, BER3	0.83	0.69	0.80	0.72	0.89	0.77	0.89	0.89
BER3, BER4	0.83	0.67	0.78	0.60	0.90	0.73	0.90	0.85
BER1, BER3	0.74	0.64	0.71	0.60	0.86	0.74	0.88	0.82
BER1, BER4	0.76	0.67	0.79	0.59	0.89	0.81	0.93	0.85
BER2, BER4	0.88	0.85	0.86	0.76	0.91	0.90	0.92	0.90

LNG1, LNG2	0.83	0.73	0.78	0.51	0.96	0.88	0.90	0.95
LNG1, LNG3	0.89	0.87	0.85	0.70	0.97	0.94	0.91	0.94
LNG2, LNG3	0.85	0.75	0.71	0.60	0.98	0.90	0.79	0.98

GST1, GST2	0.84	0.82	0.77	0.81	0.88	0.87	0.80	0.87
GST3, GST2	0.78	0.69	0.77	0.66	0.90	0.79	0.87	0.86
GST1, GST3	0.78	0.74	0.72	0.74	0.92	0.89	0.85	0.85

KDN1, KDN2	0.90	0.52	0.77	0.81	1.00	0.71	0.99	0.99
KDN1, KDN3	0.93	0.69	0.80	0.78	0.94	0.95	1.00	0.99
KDN3, KDN2	0.93	1.00	0.98	0.70	0.96	1.00	1.00	1.00

Average	*0.82*	0.72	0.77	0.67	*0.91*	0.83	0.89	0.89

Ranks	*61*	35	42	22	*52*	28	42	39

**Table 4:** Average scores of the top 10% of the best performing KEGG pathways for each classifier.

		ACC				AUC		
**datasets**	**BNKL**	**SVM**	**LAS**	**PC**	**BNKL**	**SVM**	**LAS**	**PC**

BRS1,BRS2	0.61	0.57	0.56	0.61	0.64	0.63	0.61	0.64

BER1,BER2	0.77	0.75	0.74	0.74	0.84	0.87	0.87	0.82
BER2,BER3	0.75	0.72	0.77	0.69	0.82	0.80	0.86	0.78
BER3,BER4	0.71	0.65	0.75	0.68	0.80	0.73	0.85	0.77
BER1,BER3	0.69	0.65	0.68	0.65	0.75	0.74	0.79	0.74
BER1,BER4	0.74	0.69	0.72	0.73	0.82	0.79	0.85	0.80
BER2,BER4	0.83	0.81	0.81	0.76	0.88	0.89	0.89	0.83

LNG1,LNG2	0.77	0.76	0.78	0.82	0.92	0.92	0.92	0.91
LNG1,LNG3	0.90	0.90	0.86	0.83	0.94	0.95	0.91	0.89
LNG2,LNG3	0.81	0.80	0.81	0.78	0.91	0.95	0.93	0.87

GST1,GST2	0.82	0.78	0.85	0.75	0.87	0.88	0.88	0.83
GST3,GST2	0.78	0.79	0.80	0.71	0.85	0.89	0.89	0.79
GST1,GST3	0.82	0.77	0.82	0.71	0.90	0.88	0.92	0.80

KDN1,KDN2	0.89	0.85	0.82	0.80	0.98	0.97	0.95	0.96
KDN1,KDN3	0.89	0.82	0.86	0.76	0.98	0.97	0.99	0.97
KDN3,KDN2	1.00	1.00	0.99	0.82	1.00	1.00	0.99	0.99

Average	**0.80**	0.77	0.79	0.74	0.87	0.87	**0.88**	0.84

Ranks	**53**	35	47	25	46	42	**50**	22

**Table 5:** Classifier comparison based on ACC differences over all tested pathways.

GSE data sets	BNKL-SVM	BNKL-LAS	BNKL-PC
BRS1, BRS2	2.65 (0)	3.32 (0)	0.86 (0)

BER1, BER2	0.45 (0.08)	2.04 (0)	6.89 (0)
BER2, BER3	3.26 (0)	-1.32 (0)	10.58 (0)
BER3, BER4	6.33 (0)	-2.01 (0)	7.59 (0)
BER1, BER3	2.32 (0)	0.20 (0.14)	5.09 (0)
BER1, BER4	3.64 (0)	0.42 (0.02)	3.37 (0)
BER2, BER4	2.79 (0)	2.04 (0)	12.64 (0)

LNG1, LNG2	-1.18 (0)	-1.56 (0)	5.43 (0)
LNG1, LNG3	-1.35 (0)	4.67 (0)	12.68 (0)
LNG2, LNG3	-2.37 (0)	-0.60 (0.17)	8.47 (0)

GST1, GST2	5.23 (0)	-0.95 (.07)	20.48 (0)
GST3, GST2	-1.41 (0)	-1.47 (0)	12.37 (0)
GST1, GST3	3.77 (0)	0.27 (0.56)	15.25 (0)

KDN1, KDN2	2.17 (0)	3.72 (0)	23.98 (0)
KDN1, KDN3	5.06 (0)	2.34 (0)	22.87 (0)
KDN3, KDN2	-1.72 (0)	0.90 (0)	31.27 (0)

Shown are the median differences and, in parentheses, the Mann-Whitney test p-values (those less than 0.01 are set to 0).

$$\begin{array}{c} ACC=\left(n_{B}ACC_{A\to B}+n_{A}ACC_{B\to A}\right)/\left(n_{A}+n_{B}\right)\\ AUC=\left(n_{B}AUC_{A\to B}+n_{A}AUC_{B\to A}\right)/\left(n_{A}+n_{B}\right). \end{array}$$

### Class prediction using differentially expressed genes

A standard practice in comparative microarray studies is to perform discrimination analysis employing biomarkers - genes manifesting differential expression between contrasting experimental conditions. A DE-based approach can be implemented by some routine statistical procedures such as linear discrimination analysis, logistic regression and, from the above discussed algorithms, SVM and LASSO. All these methods however are essentially uni-variate for they do not account for possible interactions among the biomarker genes. In contrast, the proposed BNKL method, along with PC, selects and accounts for significant gene interactions. It is an open question of whether in practice discrimination analysis actually benefits from employing interaction models. The results presented below partially address this question.

We implement the following DE-based test framework. For each training set, we identify DE genes by performing two-sample t-tests and then order the genes according to increasing p-values. Then we select the top 25, 50, 75 and 100 DE genes as features and supply them to each classifier. For more robust performance evaluation, overall ACC and AUC scores are formed by averaging the scores achieved on the above defined 4 DE sets. Since the selected genes are highly discriminating, we expect all classifiers to achieve their highest potential prediction scores. In particular, we consider the performance of LASSO to be representative of what would be the best prediction accuracy of a routine biomarker approach.

Table 3 presents the prediction scores using the top 100 DE genes as described. Listed are ACC and AUC for each data set pair as well as the overall average scores and total ranks. The latter are obtained as follows. For each data set pair (table row) the classifiers are ranked from 1 (lowest) to 4 (highest) according to their scores and then the ranks in each column are summed to obtain the total ranks. In terms of ACC, BNKL most often achieves best accuracy and has the best total rank of 61, followed by LASSO with rank 42. With respect to AUC, the difference between BNKL and LASSO is similarly prominent, rank 52 vs. 42. In terms of average performance BNKL also achieves the best ACC and AUC scores. These results clearly indicate the potential value of incorporating BNKL in a biomarker framework.

### Pathway-based classification

In the field of systems biology, pathways have been introduced as means for linking the functionality of groups of genes to specific biological processes. Well established methodologies such as Gene Set Enrichment Analysis (GSEA) [40], employ pathways as functional units to differentiate between experimental populations. In the context of CBN learning, we utilize pathways as priors to facilitate inference and lessen the computational complexity. First, BNKL learning benefits from the limited number of genes in the pathways.

Second, since the genes in the pathways are putatively related, it is reasonable to presume class differences in their interactions. Note that when no significant interactions are detected, BNKL is essentially equivalent to a naive classifier.

In this second validation scenario, we consider a collection of manually curated pathways based on expert knowledge and existing literature obtained from the Kyoto Encyclopedia of Genes and Genomes (KEGG, http://www.genome.jp/kegg/pathway.html). To limit the computational cost, we consider only pathways of size less than 400. The resulting collection contains 225 gene pathways of variable size, from 10 to 389. We apply BNKL and the benchmark classifiers on each selected pathway and record the achieved ACC and AUC scores. Since for a particular sample phenotype or disease condition only limited number of genes may show expression activity, we cannot expect all pathways to perform equally well in terms of prediction power. We therefore propose to select the top 10% of the best performing pathways for each classifier and report their average prediction scores.

Table 4 shows the prediction scores for each sample pair and the average score and total rank of each classifier. The BNKL classifier achieves the highest overall ACC score of 0.80. On the other hand, the AUC score of 0.87 for BNKL is slightly lower than LASSO's 0.88, not significantly so however as we show in the comparison tests below. We thus conclude that the pathway-based performance of BNKL and LASSO, unlike the DE scenario, are similar.

In Table 5 we compare the algorithms' performance in terms of ACC based on the pathway scores as follows. The pathway ACCs of the benchmark classifiers are subtracted from that of BNKL and then Mann-Whitney test is appied on each of the resulting 3 sets of 225 (the number of tested pathways) differences. A significant positive median difference indicates better performance of BNKL, a negative one favors the competing classifier. As shown, BNKL performs significantly better than SVM for 10 out of the 16 data set pairs. In the BNKL vs. LASSO comparison, BNKL is significantly better in 8 cases, while LASSO in 4. On the other end, the PC-based algorithm presents the lowest performance among the 4 classifiers.

### Comparative performance summary with discussion

In the next table we conveniently summarize the detailed results presented in Tables 3 and 4. We report the mean ACC and AUC scores along with the standard deviations in parentheses.

**Figure 2:**
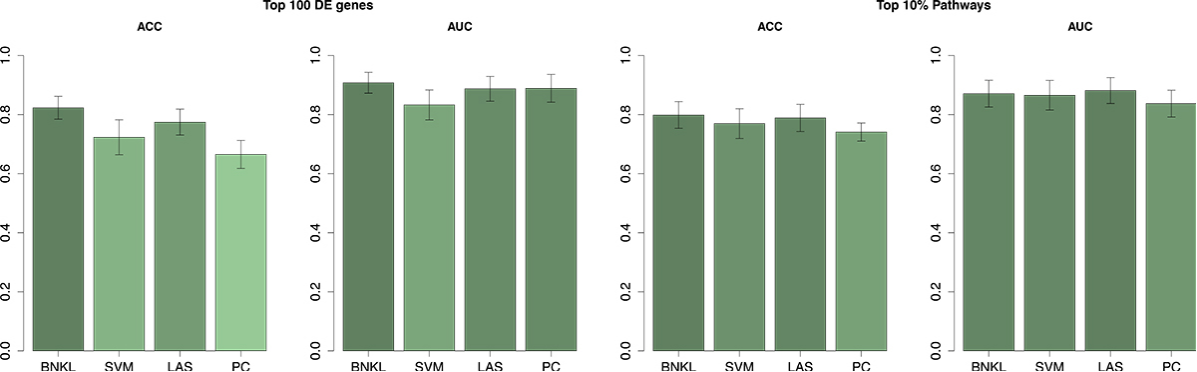
**Summary of the classification performance using DE genes and KEGG pathways**. Shown are the average ACC and AUC over the considered data set pairs along with the standard deviation.

In addition, Figure 2 visualizes the above scores in form of bar-plots. The best overall ACC (0.82) and AUC (0.91) scores are achieved by BNKL using top 100 DE genes. Interestingly, the top 10% pathways ' scores of SVM and LASSO are slightly better than their DE-based scores.

We also perform a more formal comparison using the ACC and AUC differences between BNKL and the 3 benchmark algorithms. We subtract the scores of SVM, LASSO and PC from that of BNKL, report the median differences and, in parentheses, the p-values corresponding to Mann-Whitney tests hypothesizing equal scores. Note that positive differences are in favor of BNKL and those significant at 0.05 level are emphasized.

The above results demonstrate the strong comparative performance of BNKL especially in terms of ACC.

The prediction scores of BNKL and LASSO are in close range. It is noticeable that in the pathway scenario BNKL losses the clear performance gain it has over LASSO in the DE case. The most probable explanation of this fact is that LASSO performs an active model selection by discarding all insignificant genes from a given pathway as model covariates; and this pruning improves its prediction power. On the other hand, BNKL, although focused on choosing the most significant regulations, does not exclude from using even those genes which are found to be in no interaction with the rest. As a result, including insignificant genes in the log-likelihood (1) actually hampers the prediction power of BNKL. The problem is not observable in the DE scenario where only highly discriminating genes are used. We believe that this limitation of BNKL can and should be addressed in future versions of the algorithm. Another difference between BNKL and the other 3 methods, the effect of which is yet to be investigated in details, is due to the additional discretization step involved in BNKL. Employing more sophisticated discretization procedures that provide better representation of the marginal distributions of gene expression values is likely to improve the performance of BNKL.

As a final comment, among the 4 algorithms PC trails behind with the lowest scores, which we contribute to its model selection insufficiency - close inspection shows that PC fits too complex networks (data not shown) thus overfitting the training data and degrading its prediction performance.

### Differential regulation analysis of two types of lung cancer

In a comprehensive study [2] of squamous cell lung carcinoma (SQCC), the importance of several genes implicated in the disease condition have been reported, among which TP53, CDKN2A, PIK3CA, RAS (HRAS and KRAS), EGFR and NOTCH1. These are genes involved in cell cycle control, apoptosis and cell differentiation, and possibly express distinct alternation pattern in SQCC in comparison to adenocarcinoma, the other most common type of lung cancer. The presented below pathway-based analysis corroborates with these findings and serves as a validation of the proposed BNKL methodology.

**Figure 3:**
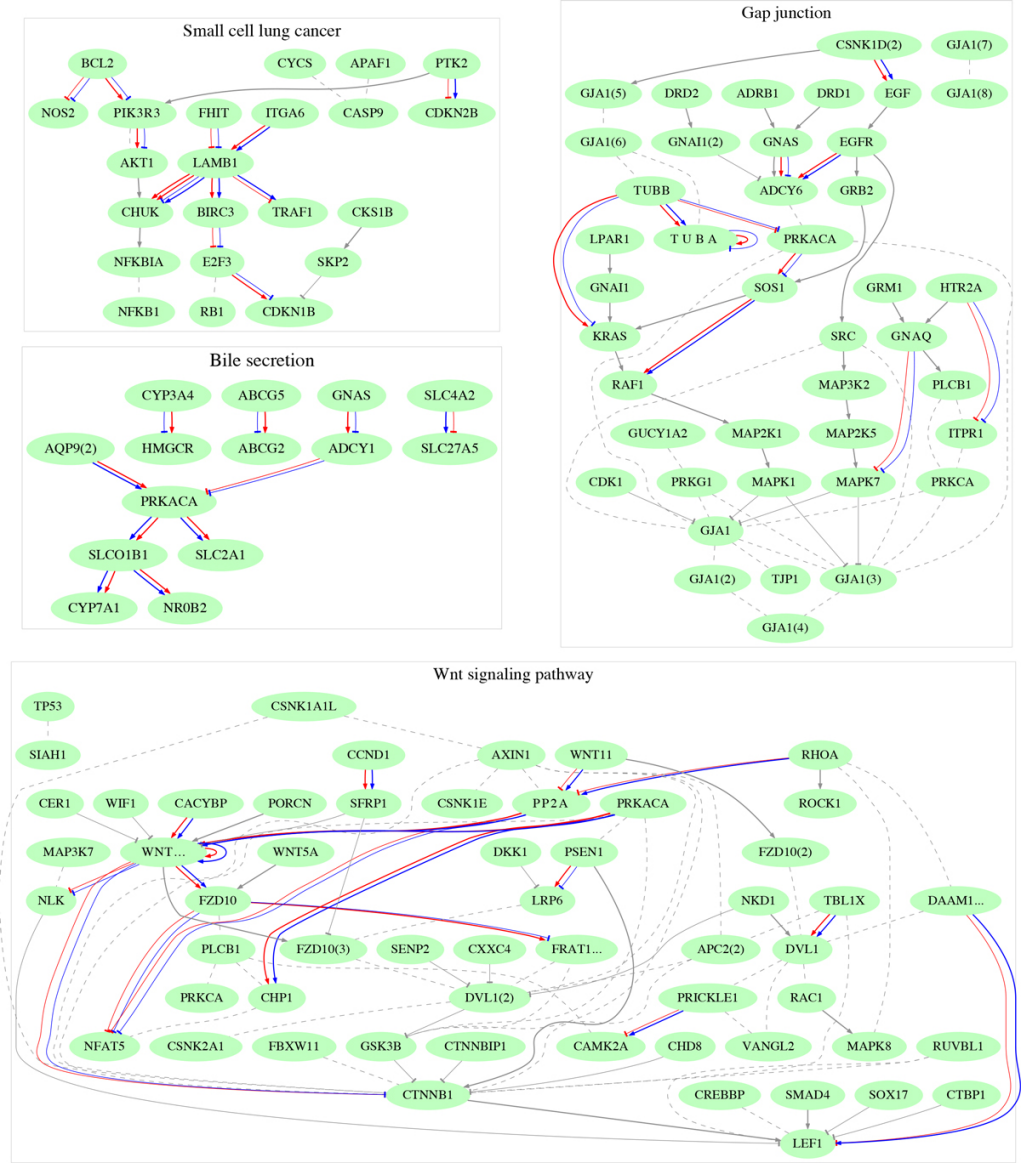
**Pathway analysis of the LNG1 data set**. Four estimated BNKL networks with edges shown in red(first class) and blue(second class) are overlaid on the corresponding KEGG pathways with edges drawn in gray. When available, also indicated are the type of regulations - activation (normal arrowhead) and inhibition (tee arrowhead).

Table 6 shows the KEGG pathway-based ACC scores for the (LNG1,LNG3) pair along with the top 16 pathways with best performance achieved by either one of the 4 classifiers. *Small cell lung cancer, Wnt signaling *and *Bile secretion *are among the best performing pathways. In Figure 3 we show some of the BNKL estimated DAGs overlaid on the original, curated KEGG pathways. The edges of the BNKL networks are color-coded in red and blue to differentiate the class regulations. For the purpose of illustration, different isoforms or versions of a gene are represented by one node, which may result in loops seemingly incompatible with the original DAGs. As seen, the BNKL networks are relatively sparse in comparison to the curated KEGG networks for, recall, only associations with significant class differences are picked up by BNKL. The plots also highlight a key feature of the presented framework - identification of differentially expressed gene regulations that reveal easy to interpret functional changes between disease conditions. We proceed with some more details.

**Table 6:** Top performing pathways by ACC prediction accuracy for the (LNG1, LNG3) pair.

Pathway	BNKL	SVM	LAS	PC
Axon guidance	0.87	**0.93**	0.83	0.70
Melanogenesis	**0.92**	0.83	0.66	0.72
Tight junction	**0.92**	0.88	0.89	0.73
p53 signaling pathway	**0.92**	0.91	0.76	0.72
Pathways in cancer	0.89	0.86	**0.91**	0.74
Complement and coagulation cascades	0.75	**0.91**	0.74	0.52
Fructose and mannose metabolism	0.82	**0.91**	0.82	0.60
Chronic myeloid leukemia	0.86	**0.91**	0.74	0.72
Bile secretion	0.90	0.87	0.72	0.67
Small cell lung cancer	0.87	**0.90**	0.71	0.69
Wnt signaling pathway	**0.90**	0.84	0.69	0.64
Cell adhesion molecules (CAMs)	0.88	**0.90**	0.67	0.53
Leukocyte transendothelial migration	**0.90**	0.89	0.81	0.54
Endocytosis	**0.90**	0.88	0.86	0.67
Non-small cell lung cancer	**0.90**	0.89	0.74	0.77
T cell receptor signaling pathway	**0.88**	0.88	0.76	0.74

First we observe that BNKL often represents indirect actual associations, connecting with directed edges genes which are at the end of regulation cascades in the curated pathways. For example, in the *Small cell lung cancer *there is a long chain of regulations connecting the ECM-receptor LAMB1 and TRAF1 which is represented by a directed edge in BNKL - inhibition in the first class (red tee arrowhead) and activation in the second (blue arrowhead). LAMB1→BIRC3 is another example of association shortcut. The edges in the *Bile secretion *pathways are mostly indirect regulations. In the *Wnt signaling *pathway the WNT16→CTTNB1 edge selected by BNKL is a shortcut for the regulation chain WNT16→FZD10→DVL1→GSK3B→CTTNB1. In other cases however, BNKL draws edges between genes which are known to interact directly such as PIK3R3→AKT1 in the *Small cell lung cancer *and GNAS→ADCY6 in the *Gap junction *pathway. As a side note, the active presence of PIK3R3 in the estimated BNKL network is in agreement with the already established characteristic role of PIK3 gene family in SQCC [41].

Next we inspect more closely the *Gap junction *pathway, which regulates intercellular communication and is involved in tumor progression. It has been reported in [42] that the expression of one of the key genes involved in this pathways, GJA1, which encodes the connexin43 protein, is reduced in human and mouse lung carcinoma cells. According to the curated KEGG pathway, tubulin-beta proteins (TUBB and TUBA) bind to connexin43 and the expression of the latter is inhibited by MAPK7. In the BNKL reconstructed network there is an indirect inhibition of MAPK7 by GNAQ which is stronger in the case of adenocarcinoma. Moreover, TUBB6 is strongly associated with TUBA1B, inhibits PRKACA and expresses differential regulation on KRAS (activation in case of adenocarcinoma and inhibition in case of SQCC) thus emphasizing the importance of the regulation changes in tubulin-beta for distinguishing the two types of lung cancer. Another notable differential interaction selected by BNKL is GNAS→ADCY6 (activation in adenocarcinoma and suppression in SQCC) while, according to KEGG, in normal cells we have a stimulating effect of GNAS, the gene encoding the G-protein, on ADCY6. An indirect association between EGFR and ADCY6 is also detected. We recall that EGFR is a recognized oncogene and is being investigated as a potential therapeutic target [41].

**Figure 4:**
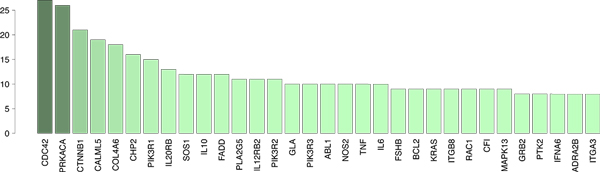
**Top 32 most connected genes from the BNKL pathway analysis of LNG1**. Connectivity is indicated on the y-axis as number of neighbors (either parents or children).

Finally we identify and report the most connected genes in the BNKL reconstructed pathways. For the purpose, we integrate all estimated KEGG pathways and for each gene we count the number of directed edges (in and out-bound) to other genes. Then we rank the genes according to thus accumulated scores to obtain the most connected ones; see Figure 4. These are genes with most marked involvement in the differentiation between adenocarcinoma and SQCC. Among them are CDC42 (cell division control protein), PRKACA (cell signaling), CTNNB1 (cell adhesion), CHP2 (cell proliferation and tumor growth), PIK3R1 (cell proliferation and survival) and KRAS (a known oncogene and potential lung cancer drug target) which play key roles in cell-to-cell signaling, as well as, cell growth, arrest and death.

## Conclusion

Many of the problems accompanying the analysis of gene expression profiles are caused by technological noise, platform and lab related bias, and small sample size. Categorical Bayesian networks mitigate some of these problems by providing noise and bias reduction through discretization, ability to handle non-linear gene interaction effects and efficient multivariate model representation. We have developed a framework for discrimination analysis, BNKL, based on the reconstruction of an optimal graph structure from two-class labeled data. The proposed score-based learning algorithm uses a KL-divergence criteria to maximize the observed class separation. The performed extensive analysis on real data has demonstrated the competitive-ness of our approach with respect to some established classification algorithms. The distinctive advantage of BNKL - its utility in discovering differentially expressed regulations between comparable conditions - has been applied for discriminating cancer sub-types. In particular, we have utilized BNKL to model the difference between adenocarcinoma and squamous cell lung cancers.

Understandably, the BNKL classifier is limited by the computation complexity of its learning algorithm and its direct application to multi-thousand gene sets can be prohibitive. In our experiments we have restrained the complexity by using manually curated pathways and subsets of differentially expressed genes. However, a whole genome analysis can be also achieved by restricting the number of allowed parents for each gene-node. Potential parents can be selected according to the degree of association with the child genes or using some prior information such as the KEGG pathway database of gene interactions. The current software realization of the algorithm [20] allows for implementation of such strategies.

We want to point to other application possibilities of BNKL beyond the microarray expression data used in this study. Next generation sequencing technologies provide an ample source of new genetic samples. For example, single-nucleotide polymorphism (SNP) samples, being genuinely discrete, can be immediately utilized. Adapting BNKL to new data modes and extending its area of application is a subject of ongoing investigation.

## Abbreviations

Bayesian Networks (BN), Categorical Bayesian Networks (CBN), Directed Acyclic Graph (DAG), Cross-Validation (CV), Maximum Likelihood (ML), Support Vector Machines (SVM), Kullback-Leibler (KL), Differential Expression (DE), Increasing Differential Expression (IDE).

## Competing interests

The author declares that there are no competing interests.

## Declarations

The publication costs of this article were funded by the corresponding author.

This article has been published as part of *BMC Medical Genomics *Volume 6 Supplement 3, 2013: Selected articles from the IEEE International Conference on Bioinformatics and Biomedicine 2012: Medical Genomics. The full contents of the supplement are available online at http://www.biomedcentral.com/bmcmedgenomics/supplements/6/S3.

## References

[B1] Kreeger PK Lauffenburger DA Cancer systems biology: a network modeling perspective Carcinogenesis 2010 6 1 2 8 10.1093/carcin/bgp261 19861649 PMC2802670

[B2] The Cancer Genome Atlas Research Network Comprehensive genomic characterization of squamous cell lung cancers Nature 2012 6 7417 519 25 10.1038/nature11404 22960745 PMC3466113

[B3] Woolf PJ Bayesian analysis of signaling networks governing embryonic stem cell fate decisions Bioinformatics 2005 6 6 741 53 10.1093/bioinformatics/bti056 15479714

[B4] Friedman N Linial M Nachman I Pe'er D Using Bayesian networks to analyze expression data J Comput Biol 2000 6 601 620 10.1089/106652700750050961 11108481

[B5] Tibshirani R Regression shrinkage and selection via the LASSO J Royal Stat Soc B 1996 6 267 288

[B6] Cortes C Vapnik V Support-vector networks Machine Learning 1995 6 273 297

[B7] Spirtes P Glymour C Scheines R Kauffman S Aimale V Wimberly F Constructing Bayesian network models of gene expression networks from microarray data Proc Atl Symp on Comp Biology 2000 1 5

[B8] Imoto S Higuchi T Goto H Tashiro K Kuhara S Combining microarrays and biological knowledge for estimating gene networks via Bayesian networks IEEE Comp Sys Bioinformatics 2003 6 104 113 16452784

[B9] Ibrahim J Chen M Gray R Bayesian Models for Gene Expression With DNA Microarray Data JASA 2002 6 457 88 99 10.1198/016214502753479257

[B10] Helman P Veroff R Atlas S Willman C A Bayesian Network Classification Methodology for Gene Expression Data J Comput Biol 2004 6 4 581 615 10.1089/cmb.2004.11.581 15579233

[B11] Heckerman D Geiger D Chickering D Learning Bayesian networks: The combination of knowledge and statistical data Machine Learning 1995 6 197 243

[B12] Spirtes P Glymour C Scheines Causation, Prediction, and Search The MIT Press 2000 2

[B13] Buntine W A guide to the literature on learning graphical models IEEE Transactions on knowledge and data engineering 2006 6 2

[B14] Braga-Neto U Fads and fallacies in the name of small-sample microarray classification IEEE Sig Proc Mag 2007 6 91 99

[B15] Shmulevich I Zhang W Binary analysis and optimization-based normalization of gene expression data Bioinformatics 2002 6 4 555 565 10.1093/bioinformatics/18.4.555 12016053

[B16] Parmigiani G Garrett E Anbazhagan R Gabrielson A statistical framework for expression-based molecular classification in cancer J Royal Stat Soc B 2002 6 4 717 736 10.1111/1467-9868.00358

[B17] Zilliox MJ Irizarry RA A gene expression bar code for microarray data Nat Methods 2007 6 911 913 10.1038/nmeth1102 17906632 PMC3154617

[B18] Balov N A discrete Bayesian network framework for discrimination of gene expression profiles Bioinformatics and Biomedicine (BIBM), 2012 IEEE International Conference on: 4-7 October 2012 2012 1 7 10.1109/BIBM.2012.6392692

[B19] Cooper G Herskovitz E A Bayesian method for the induction of probabilistic networks from data Machine Learning 1992 6 330 347

[B20] Balov N sdnet: Soft Discretization-based Bayesian Network Inference CRAN 2012 R package version 1.01.7

[B21] Friedman N Geiger D Goldszmidt M Bayesian network classifiers Machine Learning 1997 6 131 163 10.1023/A:1007465528199

[B22] Statnikov A Aliferis CF Tsamardinos I A comprehensive evaluation of multicategory classification methods for microarray gene expression cancer diagnosis Bioinformatics 2005 6 5 631 643 10.1093/bioinformatics/bti033 15374862

[B23] Kalisch M Bühlmann P Estimating high-dimensional directed acyclic graphs with the PC-algorithm Machine Learning Research 2007 6 613 636

[B24] Irizarry RA Hobbs B Collin F Beazer-Barclay YD Antonellis KJ Scherf U Speed TP Exploration, normalization, and summaries of high density oligonucleotide array probe level data Biostatistics 2003 6 2 249 64 10.1093/biostatistics/4.2.249 12925520

[B25] Pawitan Y Bjöhle J Amler L Borg AL Gene expression profiling spares early breast cancer patients from adjuvant therapy: derived and validated in two population-based cohorts Breast Cancer Res 2005 6 6 R953 64 10.1186/bcr1325 16280042 PMC1410752

[B26] Miller LD Smeds J George J Vega VB An expression signature for p53 status in human breast cancer predicts mutation status, transcriptional effects, and patient survival PNAS 2005 6 38 13550 5 10.1073/pnas.0506230102 16141321 PMC1197273

[B27] Sotiriou C Wirapati P Loi S Harris A Gene expression profiling in breast cancer: understanding the molecular basis of histologic grade to improve prognosis J Natl Cancer Inst 2006 6 4 262 72 10.1093/jnci/djj052 16478745

[B28] Desmedt C Piette F Loi S Wang Y Strong time dependence of the 76-gene prognostic signature for node-negative breast cancer patients in the TRANSBIG multicenter independent validation series Clin Cancer Res 2007 6 11 3207 14 10.1158/1078-0432.CCR-06-2765 17545524

[B29] Dedeurwaerder S Desmedt C Calonne E Singhal SK DNA methylation profiling reveals a predominant immune component in breast cancers EMBO Mol Med 2011 6 12 726 41 10.1002/emmm.201100801 21910250 PMC3377115

[B30] Wang Y Klijn JG Zhang Y Sieuwerts AM Gene-expression profiles to predict distant metastasis of lymph-node-negative primary breast cancer Lancet 2005 6 9460 671 9 15721472 10.1016/S0140-6736(05)17947-1

[B31] Kuner R Muley T Meister M Ruschhaupt M Global gene expression analysis reveals specific patterns of cell junctions in non-small cell lung cancer subtypes Lung Cancer 2009 6 1 32 8 10.1016/j.lungcan.2008.03.033 18486272

[B32] Sanchez-Palencia A Gomez-Morales M Gomez-Capilla JA Pedraza V Gene expression pro-filing reveals novel biomarkers in nonsmall cell lung cancer Int J Cancer 2011 6 2 355 64 10.1002/ijc.25704 20878980

[B33] Starczynowski DT Lockwood WW Deléhouzée S Chari R TRAF6 is an amplified oncogene bridging the RAS and NF-kB pathways in human lung cancer J Clin Invest 2011 6 10 4095 105 10.1172/JCI58818 21911935 PMC3195480

[B34] Cheng L Wang P Yang S Yang Y Identification of genes with a correlation between copy number and expression in gastric cancer BMC Med Genomics 2012 6 14 10.1186/1755-8794-5-14 22559327 PMC3441862

[B35] Cui J Chen Y Chou WC Sun L An integrated transcriptomic and computational analysis for biomarker identification in gastric cancer Nucleic Acids Res 2011 6 4 1197 207 10.1093/nar/gkq960 20965966 PMC3045610

[B36] Wu Y Grabsch H Ivanova T Tan IB Comprehensive genomic meta-analysis identifies intra-tumoural stroma as a predictor of survival in patients with gastric cancer Gut 2013 6 1100 1111 10.1136/gutjnl-2011-301373 22735568

[B37] Jones J Otu H Spentzos D Kolia S Gene signatures of progression and metastasis in renal cell cancer Clin Cancer Res 2005 6 16 5730 9 10.1158/1078-0432.CCR-04-2225 16115910

[B38] Ding Y Huang D Zhang Z Smith J Combined gene expression profiling and RNAi screening in clear cell renal cell carcinoma identify PLK1 and other therapeutic kinase targets Cancer Res 2011 6 15 5225 34 10.1158/0008-5472.CAN-11-0076 21642374

[B39] Varela I Tarpey P Raine K Huang D Exome sequencing identifies frequent mutation of the SWI/SNF complex gene PBRM1 in renal carcinoma Nature 2011 6 7331 539 42 10.1038/nature09639 21248752 PMC3030920

[B40] Subramanian A Tamayo P Mootha VK Mukherjee S Ebert BL Gillette MA Paulovich A Pomeroy SL Golub TR Lander ES Mesirov JP Gene set enrichment analysis: A knowledge-based approach for interpreting genome-wide expression profiles PNAS 2005 6 15545 15550 10.1073/pnas.0506580102 16199517 PMC1239896

[B41] Angulo B Suarez-Gauthier A Lopez-Rios F Medina PP Conde E Tang M Soler G Lopez-Encuentra A Cigudosa JC Sanchez-Cespedes M Expression signatures in lung cancer reveal a profile for EGFR-mutant tumours and identify selective PIK3CA overexpression by gene amplification The Journal of pathology 2008 6 3 347 56 10.1002/path.2267 17992665

[B42] Ruch RJ Porter S Koffler LD Dwyer-Nield LD Malkinson AM Defective gap junctional intercellular communication in lung cancer: loss of an important mediator of tissue homeostasis and phenotypic regulation Experimental lung research 2001 6 3 231 43 11293326 10.1080/019021401300053984

